# Digital Pathology Identifies Associations between Tissue Inflammatory Biomarkers and Multiple Sclerosis Outcomes

**DOI:** 10.3390/cells13121020

**Published:** 2024-06-11

**Authors:** Benjamin Cooze, James Neal, Alka Vineed, J. C. Oliveira, Lauren Griffiths, K. H. Allen, Kristen Hawkins, Htoo Yadanar, Krisjanis Gerhards, Ildiko Farkas, Richard Reynolds, Owain Howell

**Affiliations:** 1 Faculty of Medicine, Health & Life and Health Sciences, Swansea University, Swansea SA2 8PP, UK; 909381@swansea.ac.uk (B.C.); alkavineed@gmail.com (A.V.); 1914523@swansea.ac.uk (J.C.O.); lauren.griffiths@swansea.ac.uk (L.G.); 1904735@swansea.ac.uk (K.H.A.); kristen.hawkins@ndcn.ox.ac.uk (K.H.); htooydn@gmail.com (H.Y.); krisjanis.gerhards@gmail.com (K.G.); o.w.howell@swansea.ac.uk (O.H.); 2 Division of Brain Sciences, Imperial College London, London SW7 2AZ, UK; i.farkas@imperial.ac.uk (I.F.); r.reynolds@imperial.ac.uk (R.R.)

**Keywords:** digital pathology, multiple sclerosis, prognostic, progression

## Abstract

Background: Multiple sclerosis (MS) is a clinically heterogeneous disease underpinned by inflammatory, demyelinating and neurodegenerative processes, the extent of which varies between individuals and over the course of the disease. Recognising the clinicopathological features that most strongly associate with disease outcomes will inform future efforts at patient phenotyping. Aims: We used a digital pathology workflow, involving high-resolution image acquisition of immunostained slides and opensource software for quantification, to investigate the relationship between clinical and neuropathological features in an autopsy cohort of progressive MS. Methods: Sequential sections of frontal, cingulate and occipital cortex, thalamus, brain stem (pons) and cerebellum including dentate nucleus (n = 35 progressive MS, females = 28, males = 7; age died = 53.5 years; range 38–98 years) were immunostained for myelin (anti-MOG), neurons (anti-HuC/D) and microglia/macrophages (anti-HLA). The extent of demyelination, neurodegeneration, the presence of active and/or chronic active lesions and quantification of brain and leptomeningeal inflammation was captured by digital pathology. Results: Digital analysis of tissue sections revealed the variable extent of pathology that characterises progressive MS. Microglia/macrophage activation, if found at a higher level in a single block, was typically elevated across all sampled blocks. Compartmentalised (perivascular/leptomeningeal) inflammation was associated with age-related measures of disease severity and an earlier death. Conclusion: Digital pathology identified prognostically important clinicopathological correlations in MS. This methodology can be used to prioritise the principal pathological processes that need to be captured by future MS biomarkers.

## 1. Introduction

Multiple sclerosis (MS) has a complex underlying neuropathology [1,2] and a highly variable clinical presentation, making it difficult to identify the most important radiological and clinicopathological associations [3,4,5,6,7]. The use of clinical descriptors, relapsing remitting (RR)MS, secondary progressive (SP)MS and primary progressive (PP)MS, with the additional features of activity and/or disability worsening aids diagnostic consistency but lacks pathological insight and sensitivity for predicting clinical disability and response to disease-modifying therapy (DMT) [8,9]. It is also likely that cases with the same clinical phenotype have a different balance of underlying pathological features [8,9,10]. The severity of widespread neuroinflammation (quantity of activated microglia/macrophages), demyelinating lesion load, lesion activity, infiltrates at perivascular/leptomeningeal sites and neuro-axonal loss, amongst other pathological variables, ultimately determines the disease course [9,10,11,12,13,14].

Owing to the disparity between clinical phenotype and underlying pathology, quantifying the pattern and activity of interactions between individual pathological variables could provide a closer clinicopathological correlation, aid the identification of pathologically informed biomarkers and provide a more accurate prediction of both clinical progress and individual patient response to treatment, which may vary across their lifetime [8,9,10,15,16,17,18].

Traditionally, the histological analysis of the complex morphological features of MS has relied on interpretive microscopy using observer-biased, subjective scoring methods. This methodology is time consuming, generates highly variable data and is difficult to apply consistently when used to assess cell morphology and histological staining [19]. Large multi-centre case cohorts are becoming an essential requirement for the identification of clinically significant tissue biomarkers. However, the data generated from large specimen cohorts using traditional microscopy are at risk of generating highly variable data with low inter-observer consistency, resulting in unreliable clinicopathological correlations [20,21]. Digital pathology (DP) combines high-resolution scanned slides with large-scale quantitative image analysis, allowing for a deeper and broader understanding of complex diseases, such as Alzheimer’s, by comprehensively scrutinising large cohorts of cases [22]. The potential of DP to investigate large postmortem cohorts of neuroinflammatory disease, such as MS, has only recently become apparent, but the limited number of studies have already identified novel clinicopathological correlations with implications for delivering disease-modifying treatment [23,24].

Using DP rather than traditional microscopy, for the reasons outlined, is an ideal approach to investigate quantitative interactions between different pathological processes from a cohort of clinically validated cases. Here, the quantitative data were subsequently correlated with clinically important parameters to confirm the identity of meaningful, clinicopathological associations in MS. Although limited to a small cohort of MS cases, we show that DP is suitable for use in larger-scale studies designed to investigate new diagnostically important phenotypes and identify clinically relevant biomarkers essential for the development of a precision phenotype-led approach to MS treatment.

## 2. Methods

### 2.1. Tissue Resource and Accompanying Clinical and Demographic Data

The UK MS Society Tissue Bank (MSSTB), Imperial College, London, operates as a community-based, nationwide, prospective donor scheme. Each case is accompanied by detailed lifetime healthcare records reviewed and summarised by a clinician with MS expertise to report metrics, such as MS type at diagnosis; frequency of relapses in the first 2 years; time to and age of progression; time to and age of substantial disability (wheelchair); and age died. Each donor brain is sampled and examined by an MSSTB-affiliated neuropathologist, and detailed reports are produced for each case.

Samples: In this pilot study, six brain tissue blocks were dissected from thirty-five cases of MS (28 females, 7 males, all progressive MS at death; see Table 1; research ethics approval 13/WA/0292 and 08/MRE09/31). The cases were selected based on the availability of tissue blocks of interest and completeness of clinical and neuropathological summaries and to represent the typical demographics of the wider UKMSSTB collection [12]. Coronal preparations of formalin-fixed superior frontal gyrus (sampled 1 cm rostral to the temporal pole), cingulate gyrus, thalamus, occipital cortex (including the striate cortex), basal pons and cerebellum (sampled in the sagittal plain to include the white matter and dentate nucleus) were paraffin-embedded and sectioned at 7 µm. These sampled areas represent a broad range of cortical and sub-cortical structures including periventricular sites, which are frequently affected in MS. Sections from each block per case were first stained with haematoxylin and eosin (H&E) and luxol fast blue/cresyl fast violet (LFB/CFV; Sigma, St. Louis, MO, USA) as part of the standard review and reporting by UKMSSTB.

### 2.2. Tissue Staining

The immunohistochemical staining protocol methods used are as reported in [23,25]. The antigen retrieval step with 0.05% citraconic anhydride (*v*/*v*) was conducted on FFPE tissue. The subsequent immunohistochemical staining was performed using a standard avidin–biotin amplification system using a peroxidase or alkaline phosphatase enzyme-linked detection system with diaminobenzidine (DAB) or Vector Blue as the chromogen (Vector Laboratories, Newark, CA, USA). The DAB-stained slides were counterstained, dehydrated, cleared in xylene and mounted in DePex, whilst the dual-colour immunostained slides with both DAB and Vector Blue reaction product (for CD3/CD20) were rinsed in deionised water and mounted in VectaMount (Vector Labs Ltd.).

Immunohistochemistry identified epitopes in myelin (mouse anti-Myelin Oligodendrocyte Glycoprotein (MOG), clone Y10, in house), microglia/macrophages and other antigen-presenting cells (mouse anti-Human Leucocyte Antigens -DP -DQ -DR (HLA), cr3/43; Dako, Glostrup, Denmark), post-mitotic neurons (mouse anti-HuC/HuD, clone 16A11; Invitrogen, Waltham, MA, USA), T cells (rabbit anti-CD3, Agilent Technologies, Santa Clara, CA, USA) and B cells (mouse anti-CD20, clone L26, Agilent) to allow for the identification and quantification of tissue architecture and demyelinated lesions, parenchymal and connective tissue microglia/macrophages, neurons and lymphocytes. Each antibody stain was run as a complete experiment, and positive (using mouse or rabbit anti-glial fibrillary acidic protein as appropriate) and negative (irrelevant primary antibody) controls were included.

### 2.3. Lesion Classification

Demyelinated lesions of the white and deep grey matter were classified as active, chronic active (referred to by others as mixed active/inactive or slowly expanding lesions) or chronic inactive lesions and were determined by the relative density and distribution of HLA+ microglia/macrophages in sections sequential to those stained for myelin (LFB/CFV and MOG) [2,26]. Active lesions displayed florid infiltration of macrophages. Chronic active lesions were characterised by a hypocellular centre and a border rich with microglia/macrophages. Chronic inactive lesions displayed a hypocellular centre and a border indistinguishable from the adjacent normal tissue in terms of the density of microglia/macrophages. Cortical lesions were recorded as subpial, intracortical or leukocortical. Areas of complete remyelination (shadow plaques) were noted but not measured.

### 2.4. Digital Pathology and the Quantification of Demyelination, Inflammation and Neuron Density

Digitised image slides (40×–200× magnification, format .svs) were imported into QuPath (v0. 4; https://qupath.github.io, accessed 1 February 2024) project files for the assessment of demyelination, inflammation and neuron density.

**Figure 1:**
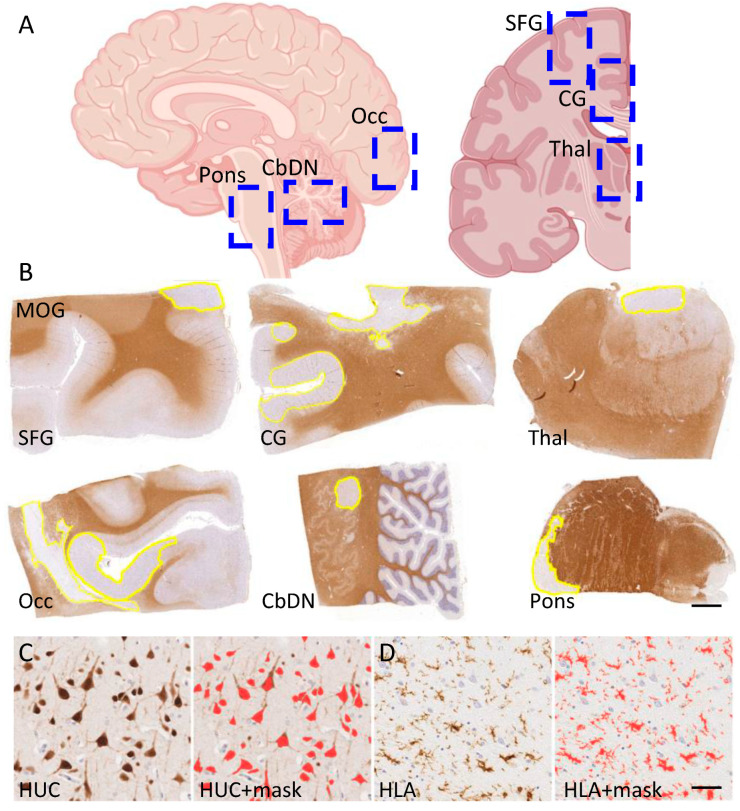
Systematic sampling of tissue blocks for immunostaining and quantification by digital pathology (**A**). Postmortem human brain tissue was sampled (**left** to **right**) at the level of the pons, cerebellum (including dentate nucleus; CbDN), occipital pole (Occ), superior frontal gyrus (SFG), cingulate gyrus (CG) and thalamus (Thal). Created with BioRender.com. Sections were prepared for immunohistochemistry, slide digitisation and quantitative pathology ((**B**); anti-MOG immunostaining to reveal areas of demyelination—yellow annotations), neuron density ((**C**); anti-HUC and red colour mask depicting automatically detected HUC+ neurons) and microglia/macrophages ((**D**); anti-HLA and red colour mask). Scale bars; (**B**) = 2 mm; (**C**,**D**) = 50 µm.

Quantification of area of demyelination within each brain area: To assess lesion area, matched LFB/CFV and MOG-stained slides were viewed in a multi-view window within QuPath. Areas of demyelination were measured by outlining the whole MOG-stained section area (cortical blocks) or anatomical site of interest (thalamus, basal pons and cerebellar white matter including dentate nucleus—see Figure 1) using the annotation tools and LFB/CFV and HUC paired slides as a reference. Total grey matter was additionally annotated for the cortical blocks. Demyelinated lesions were individually annotated, and lesions crossing anatomical boundaries (i.e., leukocortical lesions) were subdivided so that a measure of grey matter or white matter demyelination as well as total lesion area was recorded. Data were exported as a .csv file, the percent area of demyelinated lesions was calculated (Microsoft Excel), and the mean percent lesion area for cortical grey matter, thalamus, basal pons and cerebellar white matter was reported per case.

Quantification of microglial/macrophage density: The mean percent area of HLA+ immunoreactivity in normal-appearing (non-lesion) tissue was captured from each sampled block per case. The positive pixel classifier tool was used to determine the percent area of anti-HLA+ immunoreactivity in normal-appearing tissue (defined as being >10 mm from the nearest area of demyelination, whenever possible) as a measure of widespread and diffuse microglia/macrophage activation [11,27]. The area of anti-HLA+ immunoreactivity (percent of annotated area; minimum total annotation area sampled per block = 5 mm^2^) was captured, following application of the estimate stain vectors tool, through a process of colour deconvolution and optimisation parameters to differentiate background staining from immunopositive signal. The positive pixel classifier command (resolution 1.01 µm/pixel (Full); Gaussian Sigma Prefilter smoothing = 0, threshold = 0.2–0.3) was used, and the quality of detection was determined by the manual inspection before data export and handling in Excel.

Quantification of T and B cells: We selected regions of interest (ROIs) from the consecutive CD3 (T cell) and CD20 (B cell) single-immunostained slides (ROI area 0.53 mm^2^, representing a single 10× magnification field of view in QuPath). ROIs sampled perivascular spaces, with reference to the anti-MOG, CD3 and CD20 stained slides, of available thalamus, occipital pole (white matter) and pons. In each anatomical preparation, ROIs were placed in lesion and normal-appearing tissue, where present, for both the CD3 and CD20 immunostained sections. The percent area of immunostaining for either CD3 or CD20 was captured using the pixel classifier command (as described above) [28]. The percent area of CD3+ or CD20+ immunostaining of the leptomeninges overlying normal-appearing cortical grey matter or demyelinated (subpial lesion) grey matter was additionally acquired from the occipital blocks. To quantify the relative extent of T- and B-cell infiltration in the MS brain tissue, we measured the percent CD3+ or CD20+ immunostained pixels per ROI and expressed the data as an average of four ROIs per normal or lesion sample per block from three blocks per case.

Quantification of neurons: The density of HuC+ neurons was reported for regions of interest (minimum area 3mm^2^) spanning the cortical ribbon (cortical blocks), the entire annotated thalamus and basal pons and the cerebellar dentate nucleus following application of the ‘estimate stain vectors’ command and agnostic of lesion location. The ROI was placed in an identifiable sulcus, e.g., SFG Superior frontal sulcus, CG-callosal sulcus and OCC calcarine sulcus, for consistency. The density of HUC+ neurons per mm^2^ was measured using a workflow command accounting for object area (to discount HuC+ oligodendrocytes) applied across the project and following a manual inspection of the quality of the cell detections. HuC+ neuron density/mm^2^ was reported per block per case.

Measuring the relative extent of perivascular and leptomeningeal infiltration: A rating of relative leptomeningeal and perivascular cellular infiltration (0–3) for each case was generated by the review of standard histologically stained sections (LFB/CFV and/or H&E) from a minimum of six brain blocks (maximum of 12) per case, depending on availability. The rating reflected the most significant cellular infiltrate in the intact leptomeninges and/or perivascular space noted for that case. The extent of leptomeningeal and perivascular infiltration was scored semi-quantitatively as absent = 0; mild (1+, equivalent to 5–30 cells); moderate (2+, equivalent to 30–50 cells); and substantial (3+, equivalent to 50+ cells in a dense infiltrate) in accordance with previous descriptions [3,25]. There were insufficient numbers of cut sections to perform the immunostaining required to confirm if any of the 3+ rated leptomeningeal aggregates represented bona-fide B-cell follicle-like structures.

### 2.5. Data Handling and Statistical Analysis

The majority of the data was non-normally distributed (D’Agostino–Pearson normality test); consequently, non-parametric statistical comparisons were used throughout (GraphPad PRISM v10). The Mann–Whitney U test was employed for two-group comparisons (for example, when comparing age of MS onset between cases sub-grouped based on the extent of leptomeningeal/perivascular infiltration), Kruskal–Wallis with Dunn’s multiple comparison post-test for three or more groups (for example, when comparing percent area HLA+ immunoreactivity between sampled blocks) or Wilcoxon matched pairs test (for example, when comparing CD3 percent area of immunoreactivity in normal and lesion tissue from the same block). Fisher’s exact test was used to assess the independence between categorical variables (for example, when comparing the presence of active demyelinating lesions in the MS sub-groups). Associations between neuropathological variables (for example, percent area HLA+ immunoreactivity with lesion area in the thalamus) or clinical milestones (for example, between percent area HLA+ and age at death) were compared by Spearman correlation analysis, and Spearman r and *p* values were reported.

## 3. Results

### 3.1. Selecting a Representative Cohort of Progressive MS

The cohort comprised thirty-five cases of PMS (females = 28, males = 7, mean age died 53.5, range 38–98 years; Table 1). Retrospectively determined age at first symptom onset, number of relapses recorded in the first 2 years, age at progression and age at substantial disability (when the use of a wheelchair was recorded due to MS) were broadly similar to those of the wider UKMSSTB collection [16] and matched exactly in terms of mean age at death (63 years versus 63.4 years for the UKMSSTB). Supplementary Table S1 gives the cause of death, age onset of MS, age at death, presence/absence of an active lesion and grade of meningeal inflammatory infiltrates (0–3) for each case in the cohort.

Systematically sampled brain tissue blocks, encompassing cortical and deep grey matter structures frequently affected in MS, were sampled from each donor case (Figure 1A,B). Sequential cut sections were immunostained to investigate the burden of inflammatory, neurodegenerative and demyelinating pathology (Figure 1B), and a quantitative digital pathology workflow, including the quantification of neuron density and the relative area of HLA+ microglia/macrophages in the normal-appearing tissue, was applied throughout (Figure 1C,D).

### 3.2. Progressive MS Is Characterised by a Highly Variable Extent of Inflammation, Demyelination and Neuron Density

**Figure 2:**
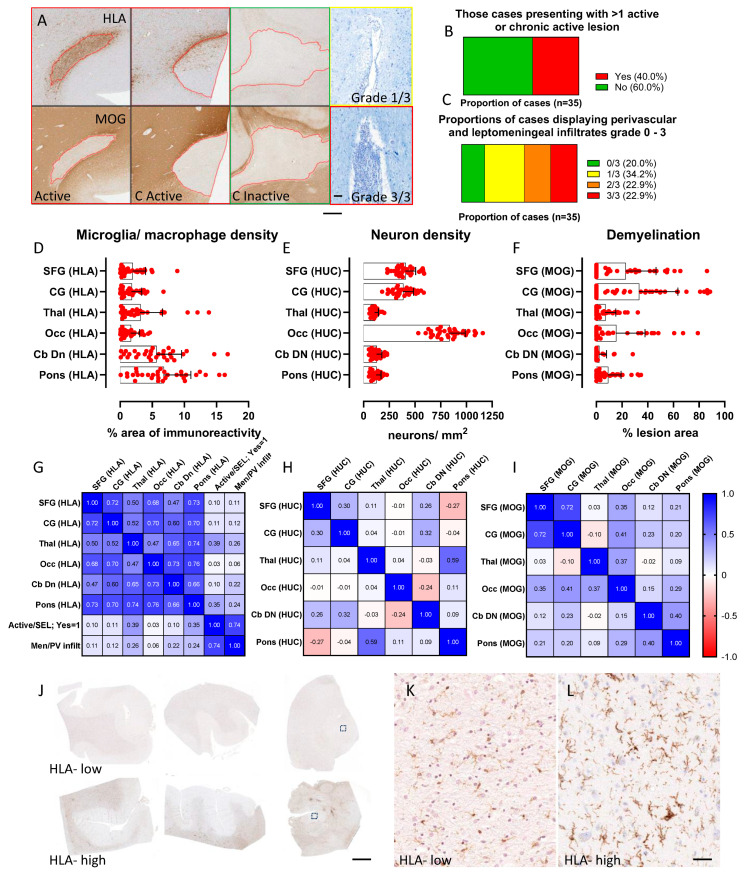
Quantifying inflammation, demyelination and neurodegeneration to reveal widespread HLA+ microglia/macrophage activation in progressive MS. Each case was further classified according to the presence/absence of active or chronic active demyelination (red image outline; (**A**)) and the relative extent of perivascular/leptomeningeal inflammation (LFB/CFV examples of infiltrates graded 1/3 and 3/3, respectively, MS330). Forty percent of cases harboured ≥1 active or chronic active lesion (**B**). Moderate (grade 2/3) or substantial (grade 3/3) levels of cellular infiltrates were noted in 45% of cases (**C**). Scatter plots representing average area of HLA + immunoreactivity (**D**), neuron density ((**E**); HUC+) and lesion area ((**F**); MOG) per region of interest, per case (group mean and standard deviation). Heat maps representing associations between microglia/macrophage density (**G**), neuron density (**H**) and lesion area (**I**) across the sampled brain regions (Spearman r values quoted). HLA+ microglia/macrophage activation in one block tended to be reflected by an elevated signal in other blocks from the same case ((**G**,**J**–**L**); representative images of SFG, CG and Thal blocks from a case with a low level of diffuse HLA+ immunosignal in comparison to a case with widespread microglia/macrophage activation; ‘HLA- high’). Images of HLA+ microglia (**K**,**L**) captured from the areas represented in the thalamus block. Significant correlations and exact p values are described in the result text. Images of HLA+ microglia (**K**,**L**)captured from areas represented in the thalamus block by dashed boxes. Abbreviations: C active, chronic active demyelinating lesion; SFG, superior frontal gyrus; CG, cingulate gyrus; Thal, thalamus; Occ, occipital pole; Cb DN, cerebellar white matter and dentate nucleus. Scale bars: (**A**) = 100 µm; (**J**) = 4 mm; (**K**,**L**) = 20 µm.

Demyelinating lesions were identified and classified based on the absence of myelin (anti-MOG immunostaining) and the relative extent of microglia/macrophage activation (Figure 2A) as active, chronic active or chronic inactive lesions. The relative extent of cellular infiltration of the perivascular and leptomeningeal spaces was recorded (Figure 2B). Forty percent (n = 14/35 cases) presented with at least one active or chronic active lesion (Figure 2C). Forty-five percent of cases (n = 16/35) presented with moderate to substantial perivascular or leptomeningeal infiltrates (Figure 2D); eight cases rated moderate (rated 2+; 22.9%); and eight cases rated with substantial (rated 3+) infiltrates.

The density of microglia/macrophages (mean percent area of parenchymal HLA+ immunoreactivity) was variable between the sampled brain areas (Figure 2E), being greatest in the cerebellar white matter (5.63%, range 0.1–16.75%) and basal pons (6.79%, range 0.08–16.30%) in comparison to cortical areas (for example, cingulate gyrus = 1.79%, range 0.007–6.74%). The mean percent HLA+ microglia/macrophage measures varied within a sampling region between cases, reflecting the heterogeneous extent of widespread microglial/macrophage activation of long-standing MS.

Neuron density (HUC+ cells/mm^2^) was captured from the grey matter areas from the same brain blocks of interest (Figure 2E). The greatest variance in neuron density was observed in the cortical blocks in comparison to blocks of thalamus, pons and dentate nucleus (for example, comparing variance between SFG and Occ HUC/mm^2^, *p* = 0.058; comparing variance between SFG and Thal HUC/mm^2^, *p* < 0.0001; F test to compare the variance of two groups).

All cases presented with at least one demyelinating lesion. The mean percent demyelination per case across all the sampled blocks ranged from 0.19 to 9.11%. Lesions were most frequently observed in the blocks comprising the basal pons (29/35 cases presented with at least one lesion in basal pons) and were less frequently observed in the block of cerebellar white matter (10/35 cases presented with at least one lesion at this site; *p* < 0.0001 when comparing the frequency of cases with at least one lesion in the basal pons in comparison to cerebellar white matter, Fisher’s exact test; Figure 2F). The mean percent lesion area was greatest in the superior frontal gyrus (22.7%, range 0–86.2%), cingulate gyrus (33.2%, range 0–88.6%) and occipital pole (15.4%, range 0–79.1%) in comparison to the cerebellar white matter (2.2%, range 0–28.4%; *p* < 0.001 for all comparisons, Kruskal–Wallis and Dunns post-test; Figure 2F).

### 3.3. The Extent of Activated Microglia/Macrophages Was Elevated across All Sampled Blocks

We investigated the inter-relationship of each pathological variable between the brain areas (Figure 2G–I). In contrast to measures of neuron density and demyelination, there was a strong inter-regional relationship between the extent of microglia/macrophage activation in the sampled blocks. For example, the mean percent HLA+ immunoreactivity in the frontal, cingulate and occipital cortical blocks was positively correlated (r > 0.68, *p* < 0.05 for all comparisons, Spearman analysis). The extent of microglial/macrophage activation was also significantly associated between the thalamus, basal pons, cerebellar white matter and occipital pole (r > 0.47, *p* < 0.05 for all comparisons; Figure 2G). The presence of active or chronic active lesions was strongly correlated with the measure of perivascular/leptomeningeal inflammation (r = 0.74, *p* < 0.00001). Active lesions and a measure of perivascular/leptomeningeal inflammation were captured per case and did not correlate with percent area of HLA captured on a per block basis (r < 0.39, *p* > 0.05, for all comparisons). The systematic sampling and analysis of brain tissue blocks allowed for us to confirm the profound microglial/macrophage activation observed in some autopsy cases, whilst others presented a low level of HLA+ signal across all areas (Figure 2J).

### 3.4. The Association between Microglial/Macrophage Activation and Other Pathological Variables Was Weak

**Figure 3:**
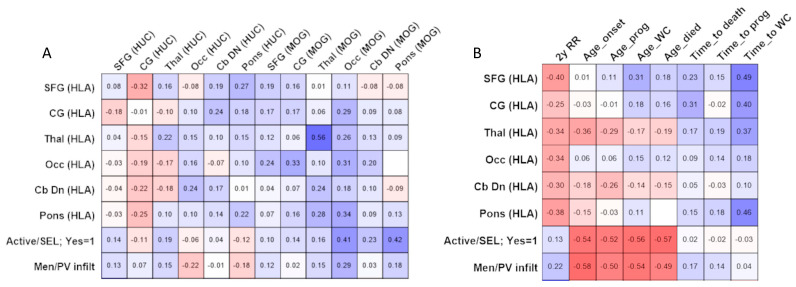
Comparing pathological variables and pathological–clinical associations. Spearman correlation analysis comparing microglia/macrophage activation in the normal appearing tissue, presence of active demyelination (Active/SEL: yes) or extent of perivascular/meningeal inflammation (Men/PV infilt; (**A**)) with neuron density (HUC) and lesion area (MOG) across the six sampled brain blocks per case. Comparing microglia/macrophage activation, presence of active lesions (Active/SEL: yes) and the relative extent of perivascular/leptomeningeal inflammation (Men/PV infilt) with clinical measures of disease severity (**B**). Note the presence of active lesions and the relative extent of perivascular/leptomeningeal inflammation with age at MS onset (Age_onset), age at secondary progression (Age_prog), age at wheelchair (Age_WC) and age died. Scale: White indicates no correlation; blue indicates positive associations; and red indicates negative associations. Individual Spearman r values are quoted for each comparison and significant correlations and exact p values are described in the results text. Abbreviations: SFG, superior frontal gyrus; CG, cingulate gyrus; Thal, thalamus; Occ, occipital pole; Cb DN, cerebellar white matter and dentate nucleus.

Spearman correlation analysis was conducted to investigate the relationship between the extent of widespread microglial/macrophage activation (percent HLA+ area of immunoreactivity) and measures of neuron density and demyelination (Figure 3A). A modest association was noted between the percent HLA+ area of immunoreactivity and lesion area at the thalamus (r = 0.56, *p* = 0.001). No other associations were noted.

There were modest correlations between lesion activity, perivascular/leptomeningeal inflammation and disease milestones (Figure 3B). For example, both active lesions and the extent of perivascular/leptomeningeal infiltrates correlated with the age of onset (*p* < 0.001), age at secondary progression (*p* < 0.004), age to wheelchair (*p* < 0.002) and age died (*p* < 0.003). Of note, neither variable associated with a shorter interval to progression, use of wheelchair or death when calculated from the age at disease onset (r = 0.172 to −0.027, *p* > 0.325, for all comparisons; Figure 3B).

### 3.5. Phenotyping Cases on the Basis of Compartmentalised Inflammation Reveals Groups That Differ by Disease Outcomes

**Figure 4:**
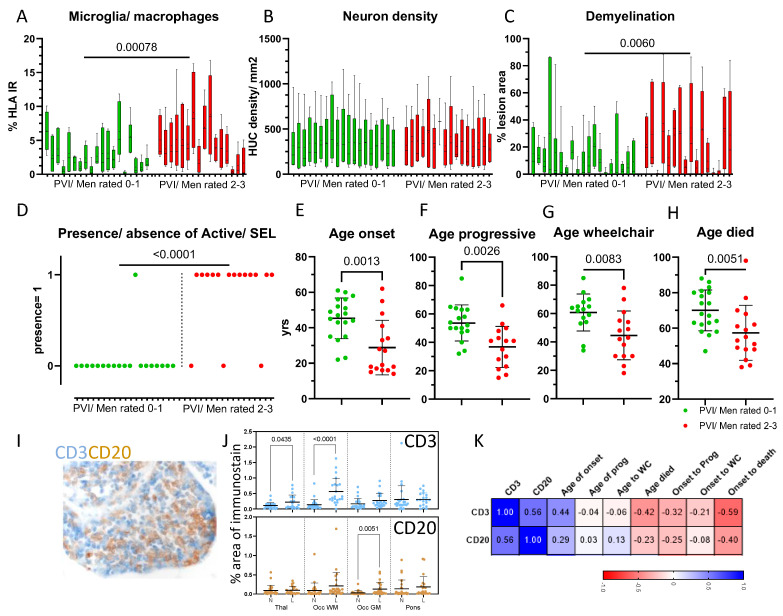
Phenotyping on the basis of the measured extent of inflammation reveals MS groups that differ by clinical outcomes. Box and whisker plots representing average percent area of HLA+ stain (**A**), neuron density (**B**) and lesion area (**C**) per case, grouped according to their relative extent of perivascular/leptomeningeal inflammation (PVI/Men rated 0–1 versus PVI/Men rated 2–3). Median, inter-quartile range and min–max values are represented. Data compared by non-parametric T-test. Cases grouped according to their relative extent of perivascular/leptomeningeal inflammation differed significantly in terms of the finding of active lesions ((**D**); Fisher’s exact test) and age at key clinical milestones ((**E**–**H**); PVI/Men rated 0–1, green data points; PVI/Men rated 2–3, red data points). Perivascular/leptomeningeal inflammation (**I**) was associated with increased density of CD3+ and CD20+ immunostaining in demyelinated thalamus, occipital pole white matter (Occ WM) and grey matter (Occ GM) in comparison to matched normal-appearing tissue (**J**). Increased CD3 and CD20+ staining associated with a younger age of death (Age died), a shorter interval to secondary progression (Onset to prog) and a shorter overall disease length (Onset to death). Spearman r values included in the heat map (**K**) and exact p values are included in the result text. Abbreviations: PVI/Men rated 0–1 or 2–3, cases grouped according to relative extent of perivascular/leptomeningeal inflammation.

We sub-grouped our autopsy cohort based on the presence of low perivascular/meningeal inflammation (termed ‘PVI/Men rated 0–1′; n = 19) or high perivascular/meningeal inflammation (‘PVI/Men rated 2–3′; n = 16 cases), as this represented the pathological variable most strongly associated with the clinical outcomes (see Figure 3B). By comparing the sub-groups, we noted a significant increase in the extent of widespread microglia/macrophage activation (*p* < 0.001, when comparing the mean percent HLA signal per case) for the ‘PVI/Men rated 2–3′ group (Figure 4A–C). Neuronal density was not different between the two groups (346.2 versus 350.0 HUC+ neurons/mm^2^, *p* = 0.859, when comparing average neuron density per case). Neuron density per block was also similar between subgroups. The average area of demyelination was greater in the ‘PVI/Men rated 2–3′ cases in comparison to ‘PVI/Men rated 0–1′ (average lesion area per block = 19.48%, range 0–88.6% and 11.06%, range 0–86.6%; *p* < 0.01).

The ‘PVI/Men rated 2–3′ group was characterised by the presence of one or more active demyelinating lesions (*p* < 0.0001 when comparing the frequency of active/chronic active lesion positive cases between sub-groups, Fisher’s exact test).

Cases stratified on the basis of their perivascular/leptomeningeal inflammation also differed in terms of their age at disease onset (mean age in years = 45.32 versus 28.81; *p* = 0.0013), age at progression (53.65 versus 36.8 years; *p* = 0.0026), age at wheelchair (60.73 versus 44.6 years; *p* = 0.0083) and age at death (70.0 versus 57.38 years; *p* = 0.0051; Figure 4E–H).

### 3.6. Meningeal Inflammation

Meningeal/perivascular infiltrates comprised variable numbers of CD3+ T cells and CD20+ B cells (Figure 4I). The density of CD3 and CD20+ immunoreactivity was quantified from perivascular and leptomeningeal sites of the thalamus, occipital pole and pons, where available (expressed as the mean percent area of CD3 or CD20 immunoreactivity per region of interest). The mean percent CD3+ area of immunostaining was elevated in lesions of the thalamus and occipital pole white matter, whilst CD20+ immunostaining was elevated in the leptomeninges overlying cortical grey matter (subpial) lesions of the occipital pole (Figure 4J). The mean density of CD3+ and CD20+ immunoreactive cells per case (when expressed as mean signal for both lesion and normal appearing across all blocks per case; Figure 4K) correlated with key clinical variables, including age of onset (CD3: r = 0.44, *p* = 0.018), age died (CD3: r = −0.42, *p* = 0.025) and disease duration (CD3: r = −0.59, *p* = 0.001; CD20: r= −0.40, *p*= 0.03), confirming the relationship that exists between tissue inflammation and disease severity.

## 4. Discussion

To develop new biomarkers that reflect the salient underlying biology of MS, we must better understand the relative contribution of pathological processes to disease outcomes. We undertook a systematic DP analysis to identify which pathological processes are most strongly associated with clinically important milestones.

The presence of diffuse inflammation (density of HLA+ microglia/macrophages in normal-appearing tissue) correlated positively between the different brain areas. These data confirm that activated microglia/macrophages, if found elevated at one site, tend to be present at elevated numbers across the MS brain. Diffuse microglia/macrophage activation, including nodules of activated microglia, is associated with axonal injury in the normal-appearing white matter [11,29]. Significant numbers of CD68+, HLA+ and complement anaphylatoxin receptor (C3a and C5a) positive cells are present in NAGM and NAWM of long-standing MS, showing that the normal-appearing tissue contains similar populations of activated microglia/macrophages but at lower densities than in focal lesions [2,23,25]. Positron emission tomography (PET) studies using radioligands to targets enriched in microglia/macrophages have confirmed the early and widespread increased density of microglia/macrophages in NAWM, which strongly associated with clinical progression but was independent of early relapses [30,31,32]. The observed elevated HLA+ microglia/macrophage signal observed across systematically sampled sites in our study suggests that white matter, deep grey matter and brain stem loci may be equally valuable sites to assess diffuse inflammation in the MS brain.

Of the systematically sampled forebrain and brainstem blocks included in our study, it was only in the thalamus that diffuse microglial/macrophage activation correlated with the relative extent of demyelination. Previous quantitative studies of clinical and autopsy cohorts have highlighted pathology of the thalamus to be a key measure of disease severity and prognosis [23,33,34]. Pathologically, elevated diffuse microglial/macrophage activation is linked to a profound activation of complement and substantial neuron loss [23], suggesting that fluid markers of innate immune activity, including complement, could be a valuable accompaniment to radiology in identifying those most at risk of disease progression [23,25,34].

As previously described, a substantial proportion of progressive (PP and SP) MS cases at postmortem that died following a prolonged disease course harboured evidence of active demyelination and/or substantial immune cell infiltration of the perivascular and leptomeningeal spaces [35]. The presence of an active lesion was positively correlated with compartmentalised (meningeal/perivascular) inflammation, and both variables correlated inversely with age at key clinical milestones, such as age to secondary progression and age to substantial disability (reported as time to wheelchair due to MS). The finding of inflammatory activity in such a high proportion of cases and the association between these variables and the attainment of moderate to substantial disability suggest that some patients with longstanding disease might still benefit from immunomodulatory treatments that are able to enter the CNS to preserve quality of life [16,17,18]. Current MS treatment protocols may not capture all those MS patients, particularly those older individuals with long-standing disease or substantial disability, who might otherwise still benefit from immunomodulation. This further highlights the need for more information about specific clinicopathological associations to help identify clinical subgroups, an example of precision clinical phenotyping [8]. There is less evidence for a treatment response in older people with MS as many clinical trials have an upper age limit of 50 years of age. Nevertheless, real world data registries and some clinical trials that included older people have shown a comparable treatment response in those older individuals still experiencing active disease [17,36].

Several studies show a moderate positive association between T2 or gadolinium-enhancing MRI lesions and long-term disability [37], while others found that the addition of new MRI-detected lesions after onset did not correlate with long-term disability [38,39]. One explanation for the apparent lack of correlation between the presence of MRI-detected lesions and long-term clinical disability includes the failure to consider concomitant grey matter lesions and meningeal inflammation [40]. Cortical, particularly subpial, and meningeal pathology are poorly resolved by current neuroimaging [41,42] but are well-recognised components of a worsening MS [14,40]. Our data are in broad agreement with the work of others in that cortical blocks displayed the most extensive relative area of demyelination, where subpial lesions overwhelmingly represent the single largest cortical lesion type and where meningeal inflammation is associated with lesion activity, microglial/macrophage density, demyelination and disease progression topography of demyelination and neurodegeneration [11,14,43,44].

In the future, it would be important to identify validated CSF and serum biomarkers that represent individual components of brain injury in MS. Serum neurofilament light-chain levels in MS are regarded as a marker of neuron–axonal injury and correlate modestly with radiological lesion volume (only in individuals with gadolinium-enhancing lesions) and subsequent loss of brain and spinal cord volume [45,46,47,48]. Glial cell and innate immune activation are partly represented by levels of Chitinase-3-like1-protein (CHI3L1). CHI3LI is associated with increased clinical severity and spinal cord volume loss [45,49]. The presence of reactive astrocytes, which are incompletely reflected by glial fibrillary acidic protein (GFAP) expression, are a pathological hallmark of MS. Clinically, CSF and serum GFAP levels correlate modestly with progression, independent of relapse activity [47,50]. We would seek to include an assessment of axonal injury and astrocyte reactivity in future DP studies.

Using digital pathology to identify biological classifiers of multiple sclerosis.

Meningeal inflammation, with or without B cell follicle-like structures [13,14,42,51], is associated with underlying neurodegeneration, microglial activation and subpial demyelination. Compartmentalised inflammation is potentially relevant to the subtle accumulation of neurological disability, independent of relapse, termed either progression independent of relapse activity (PIRA) or silent progression [39,42]. In our analysis, we found compartmentalised inflammation to be associated with the age of attaining important clinical milestones and correlated with more microglia and demyelination. By splitting our MS cohort based on the relative extent of compartmentalised inflammation, we show the association between the extent of leptomeningeal and perivascular infiltration with diffuse HLA activation and demyelination as might be anticipated. MS subgroups, defined based on their relative extent of perivascular/meningeal inflammation, also significantly differed in terms of their age of disease symptom onset and age to moderate and substantial disability and died, on average, 12 years earlier. These findings support efforts to uncover better biochemical and radiological biomarkers of this intrathecal compartmentalised response [14,15,52,53,54].

Digital pathology identifies biomarkers with implications for precision clinical phenotyping.

Classifying MS using subjective clinical descriptors does not reflect the heterogenicity of the underlying pathological process (inflammation, demyelination and neurodegeneration) and biological factors (genetic, age, gender) [8,9]. It is not surprising that individuals with the same clinical phenotype demonstrate a highly variable response to treatment because the clinical features are determined by a different balance and effect size of the principal underlying pathological and biological processes [8,9]. The variability of MS phenotypes is further influenced by age-related changes and fluctuation in the activity of pathological processes over time [3,55]. Younger people are more likely to be diagnosed with RRMS and typically experience a more slowly evolving disease than MS onset in older people. Progressive-onset MS is associated with fewer new active demyelinating lesions and inflammatory attacks and a disease that responds less well to current therapies [16,17,18]. Using DP methodology, we identified perivascular/meningeal inflammation, indicated by a variable extent of T- and B-cell infiltration, correlated with a shorter and more severe disease course [14,28,35]. Meningeal infiltrates of lymphocytes and aggregates rich in T and B cells have been reported in cortical biopsies and from acute cases of MS who died very shortly following symptom onset [56]. Meningeal inflammatory T- and B-cell infiltrates at this early stage, irrespective of clinical descriptor, are associated with the extent of demyelination and microglial activation and a younger age of attaining clinical milestones. In a subset of MS cases, meningeal inflammation was identified as a biomarker of disease progression, thus satisfying some of the clinicopathological criteria needed to deliver precision clinical phenotyping [8].

## 5. Limitations

This study is somewhat limited by the cohort size, and in the future, larger cohorts would allow for more precise definition of the clinicopathological corelates of MS. Although spinal cord pathology is a strong determinant of MS disability [57], an analysis of spinal cord was not included in the sampling strategy, owing to the limited number of cases with available spinal cord. Remyelination is a key feature of MS, and remyelination is greatest in those longest-lived cases at autopsy [58]. Remyelinating lesion area was not routinely recorded in this study, and understanding the relative contribution of remyelination with respect to the burden of tissue damage and overall disease progression is warranted. The contribution of microglia to inflammation is better assessed by reporting a panel of homeostatic and damage-associated phenotypic markers providing a more detailed correlation with clinicopathological criteria [59]. We reported neuron density as an index of relative neurodegenerative pathology. Other markers of central neurodegeneration, including synapse and neurite density, can also be routinely collected from well-preserved, formalin-fixed tissues [60]. In addition, we did not compare our findings in MS with a non-neurological control population as our study was focussed on the pathological variables that are most changed in MS and which best associate with disease outcome. Nevertheless, neuron and microglia cell density determination in suitably matched controls would have benefitted this work.

## 6. Conclusions

To mitigate the unreliability of traditional observer evaluation of neuropathology, we have developed an efficient and user-friendly DP workflow capable of reporting quantitative changes in separate pathological processes in a cohort of clinically validated cases of progressive MS.

DP has successfully handled morphological data from large cohorts of postmortem cases of neurodegenerative disease [19,22], identifying previously undetected phenotypes, ref. [61] and could be important, for example, in the identification and validation of genetic determinants of disease severity. In our study, we confirmed a central role for measures of lesion activity and cellular infiltration as key features of the compartmentalised inflammatory response that most strongly associated with clinically meaningful, age-dependant milestones of disease progression. Using DP across large cohorts could reveal the principal pathological confounders that differentially drive disease worsening, which would aid biomarker discovery to improve patient phenotyping and direct therapeutic management.

## Figures and Tables

**Table 1:** Clinical and demographic characteristics of the progressive MS cases used in this study. A detailed list is provided in Supplementary Table S1. Median (and range) quoted in years. Column headings: 2yr-RR, number of relapses recorded in first 2 years of disease; Age Prog, age at secondary progression (where applicable); Age WC, age at wheelchair; Disease Duration, time from first symptom onset to death; Age Died, average age at death for cases in the cohort.

n	Sex (Female)	Onset	2yr-RR	Age Prog	Age WC	Age Died	Disease Duration
35	28	41 (14–62)	1 (0–7)	48 (15–85)	54.5 (18–85)	63 (38–98)	27 (3–49)

## Data Availability

Upon reasonable request from the corresponding author and Owain Howell.

## Supplementary Materials

The following supporting information can be downloaded at: https://www.mdpi.com/article/10.3390/cells13121020/s1. Table S1. Sex (female = 0, male = 1), age of first MS symptom onset, age died (years), cause of death (as recorded on the death certificate, if available).
