# Supplementary material for: Digital Pathology Identifies Associations between Tissue Inflammatory Biomarkers and Multiple Sclerosis Outcomes
Source: Cells. 2024 Jun 11;13(12):1020. doi: 10.3390/cells13121020 (PMC11201856; doi:10.3390/cells13121020)
Supplement: Supplementary file 1 [file cells-13-01020-s001.zip › cells-2990402-supplementary.pdf]

Supplementary table S1:

Case	Sex	Onset	Died	Cause of death	Active lesion (y/ n)	Infiltrate (0- 3)
MS155	0	43	80	Small bowel obstruction, pleurisy, heart problem, MS	0	0
MS168	0	58	88	Bronchopneumonia	0	0
MS216	0	48	58	Breast cancer	0	0
MS247	0	60	67	MS, respiratory failure	0	0
MS312	0	45	68	Urinary Sepsis, MS	0	0
MS381	0	43	80	MS	0	0
MS602	0	61	74	Chest infection	0	0
MS086	0	51	81	Cancer of the bladder, chest infection, MS	0	1
MS288	0	56	83	Bronchopneumonia, immobility, MS	0	1
MS297	0	44	58	MS	0	1
MS306	1	35	78	Clostridium difficle diarrhea, MS	0	1
MS319	0	52	63	Carcinomatosis, metastatic bladder cancer	0	1
MS364	0	22	56	Bronchopneumonia, MS	1	1
MS410	0	33	47	MS	0	1
MS412	1	47	74	Pneumonia, chronic lymphatic leukemia, MS	0	1
MS558	0	23	62	-	0	1
MS589	0	35	64	-	0	1
MS607	0	49	63	-	0	1
MS681	0	56	86	-	0	1
MS325	1	48	51	Bronchopneumonia	1	2
MS340	0	33	53	Sepsis, aspiration pneumonia, MS, perinephric abscess	1	2
MS363	1	15	42	MS, respiratory failure	1	2
MS389	0	27	55	MS, Multiple urinary sepsis	1	2
MS406	1	14	62	Chest infection, aspiration pneumonia, MS	1	2
MS483	0	41	49	Bronchopneumonia, aspiration pneumonia, MS	1	2
MS541	0	18	68	MS	0	2
MS680	0	62	98	MS	0	2
MS103	0	55	77	Pneumonia	1	3
MS157	0	17	39	MS	1	3
MS191	0	16	48	Pneumonia, MS	0	3
MS317	0	18	48	Aspiration pneumonia due to MS	1	3
MS330	0	19	59	Pneumonia, MS	1	3
MS510	0	16	38	Pneumonia, MS	1	3
MS601	1	34	70	-	1	3
MS604	1	28	61	-	1	3
**N=35**	**7 males**	**41**	**63**	**n/a**	**0**	**1**

Supplementary table S1: Sex (female= 0, male= 1), age of first MS symptom onset, age died (years), cause of death (as recorded on the death certificate, if available). Active lesion (y/ n) indicates presence (y) or absence (n) of an active or chronic active lesion; Infiltrate (0- 3), indicates relative extent of the largest leptomeningeal and/ or perivascular infiltrate observed. Number of cases and median values for each variable are presented in the final row. n/a= not applicable; -= data not available.
